# Definition and Classification of Postoperative Complications After Cardiac Surgery: Pilot Delphi Study

**DOI:** 10.2196/39907

**Published:** 2022-10-12

**Authors:** Linda Lapp, Matt-Mouley Bouamrane, Marc Roper, Kimberley Kavanagh, Stefan Schraag

**Affiliations:** 1 Department of Computer and Information Sciences University of Strathclyde Glasgow United Kingdom; 2 Usher Institute College of Medicine and Veterinary Medicine University of Edinburgh Edinburgh United Kingdom; 3 Department of Mathematics and Statistics University of Strathclyde Glasgow United Kingdom; 4 Department of Anaesthesia and Perioperative Medicine Golden Jubilee National Hospital Clydebank United Kingdom

**Keywords:** Delphi study, cardiac surgery, postoperative complications, morbidity, postoperative, cardiology, postoperative, surgery, complications, cardiac, health services, society, pilot, development, system, surgeons, anesthetists, clinical, quality, resources, risk management, communication, research

## Abstract

**Background:**

Postoperative complications following cardiac surgery are common and represent a serious burden to health services and society. However, there is a lack of consensus among experts on what events should be considered as a “complication” and how to assess their severity.

**Objective:**

This study aimed to consult domain experts to pilot the development of a definition and classification system for complications following cardiac surgery with the goal to allow the progression of standardized clinical processes and systems in cardiac surgery.

**Methods:**

We conducted a Delphi study, which is a well-established method to reach expert consensus on complex topics. We sent 2 rounds of surveys to domain experts, including cardiac surgeons and anesthetists, to define and classify postoperative complications following cardiac surgery. The responses to open-ended questions were analyzed using a thematic analysis framework.

**Results:**

In total, 71 and 37 experts’ opinions were included in the analysis in Round 1 and Round 2 of the study, respectively. Cardiac anesthetists and cardiac critical care specialists took part in the study. Cardiac surgeons did not participate. Experts agreed that a classification for postoperative complications for cardiac surgery is useful, and consensus was reached for the generic definition of a postoperative complication in cardiac surgery. Consensus was also reached on classification of complications according to the following 4 levels: “Mild,” “Moderate,” “Severe,” and “Death.” Consensus was also reached on definitions for “Mild” and “Severe” categories of complications.

**Conclusions:**

Domain experts agreed on the definition and classification of complications in cardiac surgery for “Mild” and “Severe” complications. The standardization of complication identification, recording, and reporting in cardiac surgery should help the development of quality benchmarks, clinical audit, care quality assessment, resource planning, risk management, communication, and research.

## Introduction

The use of risk prediction tools in cardiac surgery is predominantly focused on the risk of mortality [1]. In the United Kingdom, the mortality rates after all cardiac surgery are some of the lowest in the world despite increasing age, risk profile, and frailty of patients [2]. Complications after surgery, however, are common [3,4] and, depending on severity, can have a debilitating impact on patients’ quality of life [5], increase hospital length of stay [6], and hence increase health care costs [7,8]. It is therefore essential that efforts should be “directed to further reducing morbidity and length of stay” [2] and that adequate systems are developed to better predict, anticipate, plan, and mitigate the risks for severe surgical complications. Although efforts are made to preempt postoperative complications in cardiac surgery using various technologies [9-11], the lack of a consensual and standard definition and classification of postoperative complications in cardiac surgery, however, acts as an important barrier to developing adequate monitoring and reporting systems for cardiac surgery complications [12].

This pilot study aimed to address this issue by using the Delphi method [13] to answer the following research questions:

1. What are domain experts’ opinions on the usefulness of a definition and classification of surgical complications following cardiac surgery?
2. How do domain experts define what events constitute surgical complications following cardiac surgery?
3. How do domain experts classify surgical complications following cardiac surgery?

## Methods

### Ethical Statement

This study (Health Research Authority REC18/YH/0366) was approved by the University of Strathclyde Department of Computer and Information Sciences Ethics Committee (ID 837).

### The Delphi Method

The Delphi method is a well-established expert consultation method building on the premise that group opinion is more valid and reliable than individual opinion that can be heavily influenced by cognitive bias [13]. The Delphi method uses a multistaged survey system that can be used to reach expert consensus on complex topics and loosely defined concepts and to conduct forecasting or horizon scanning [14].

**Figure 1:**
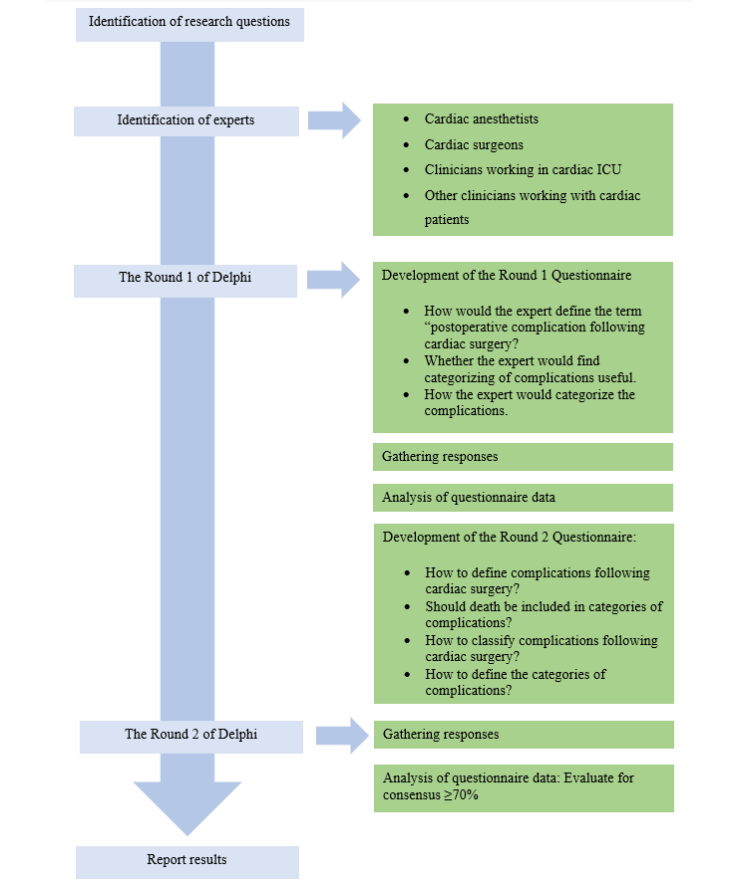
Delphi study process. ICU: intensive care unit.

The original Delphi method, also known as the Classical Delphi, consists of 2 or more rounds of questionnaires administrated by mail to an expert panel. Round 1 focuses on the experts’ opinions in an open-ended manner. After analyzing Round 1, Round 2 asks the experts to rank the statements or questions according to the opinions stated in the previous round. Rounds continue until consensus is reached on some or all questions. [13] This study used the e-Delphi method, which is a similar process to the Classical Delphi but administered as an online web survey [13]. The overall study process is outlined in Figure 1.

To guarantee experts’ anonymity in the study, the experts remained anonymous in both rounds, meaning the participants’ responses in Round 1 and Round 2 were not linked. This decision was done due to choosing the “all-rounds” approach, in which potential participants are invited to take part in subsequent rounds regardless of whether they participated in the previous rounds. It has been shown that this approach can improve representation of opinions and can reduce the chances of false consensus. [15]

### Identification of Experts

Cardiac surgery experts were identified as follows: cardiac anesthetists, cardiac surgeons, and anesthetists specializing in working with cardiac patients perioperatively or in intensive care. Since this was a pilot study to develop a definition and classification for postoperative complications in cardiac surgery, mailing lists of the following professional associations were used to invite prospective participants to the Delphi study: *Association for Cardiothoracic Anaesthesia and Critical Care*, *European Association of Cardiothoracic Anaesthesiology and Intensive Care*, *The Society for Cardiothoracic Surgery*, and *The UK Society for Computing and Technology in Anaesthesia*. Through these avenues, the invitation was sent to thousands of potentially eligible participants depending on the number of members in each society. In addition to these methods, cardiac anesthetists and cardiac surgeons in 3 Scottish cardiac centers were contacted directly via email: Golden Jubilee National Hospital, Royal Infirmary of Edinburgh, and Aberdeen Royal Infirmary (64 potential participants, 27 of them cardiac surgeons and 37 of them cardiac anesthetists).

### Methods of Analysis

The survey questionnaires were provided in English, and the data from the questionnaires were exported from Qualtrics [16] and stored in a Microsoft Excel spreadsheet. R version 4.1.1. [17] and NVivo version 12 [18] were used for quantitative and qualitative analyses, respectively.

#### Consensus

The consensus level was determined to be 70%, similar to other related studies in health research [19-21]. Descriptive statistics were used to analyze the experts’ opinions, using frequencies of responses for questions that were not open-ended. If the frequency was 70% or higher, the experts were deemed to have reached consensus on this particular response.

All responses were considered in the analysis; however, consensus was calculated based on how many experts answered each question. Partially filled responses were also included, as other published studies have done in the past [22,23].

The strategy for an event of nonconsensus was to critically evaluate and discuss the respondents’ answers and to revise the questions in the subsequent rounds.

#### Qualitative Analysis

Round 1 of the study largely included open-ended questions to determine a variety of ways the experts would choose to define and categorize complications following cardiac surgery. The thematic analysis framework [24] was used to analyze the responses to the open-ended questions, and the results were included as options for responses in the subsequent round of the study as in the Delphi method [25].

A sample of the data was coded separately by 3 researchers to ensure coding coherence and consistency. Once coding consistency was established through the initial sample coding, data coding was conducted by 1 researcher (LL). Coding consistency and thematic analysis were subsequently discussed, and conflicts were resolved at regular meetings of the study investigative team, which includes substantial expertise in mixed methods and qualitative research (MMB).

Following the guidance of Hasson et al [25], statements that were identified as identical or similar were grouped as common concepts. Once specific themes were created, the statements within a thematic group were synthesized into a single summary statement after discussion between the study investigators. The wording was kept as close as possible to the statements that had been provided by the experts. Any unique statement provided by the experts with no related statement was kept as worded originally and included directly in Round 2.

## Results

### Delphi Study Round 1

For Round 1, the questionnaire was designed to explore the experts’ general opinions regarding the definition of “postoperative complication following cardiac surgery” and categorizing postoperative complications.

The questionnaire (see Multimedia Appendix 1) started with a filter question to make sure that only eligible experts would be included in the study: “Are you in any way involved with cardiac surgery patients? (Can be preoperatively, intra-operatively and/or postoperatively).” If the answer to the question was “no,” the participant was directed to the end of the survey.

The questionnaire consisted of 3 parts: (1) the background of the expert; (2) how the expert would define the term “postoperative complication following cardiac surgery”; and (3) whether the expert would find categorizing of complications useful, and, if yes, how the expert would categorize the complications.

The data in this study were collected through online questionnaires via Qualtrics [16]. The Round 1 questionnaire was sent out twice to professional societies and to other potential experts between August 27, 2019, and September 24, 2019. In total, the Round 1 questionnaire was open for 6 weeks and closed on October 8, 2019.

### Expert Demographics

Overall, 71 experts were eligible to take part in Round 1 of the study based on being involved with a cardiac surgery patient pathway. The majority (67/71, 94%) of the respondents were based in the United Kingdom, 2 (2/71, 3%) were from Saudi Arabia, 1 (1/71, 1%) was from Australia, and 1 (1/71, 1%) was from Bahrain.

Most of the respondents (45/71, 63%) specialized in both cardiac anesthesia and cardiac critical care, 23 (23/71, 32%) specialized in cardiac anesthesia only, and 3 (3/71, 4%) specialized in cardiac critical care only. It is important to note that none of the participants stated that they were cardiac surgeons. This is further discussed in the *Limitations* section. In terms of experience, the mean number of years worked in the specialty was 16.63 (SD 8.70) years, and the median number of years was 16 (IQR 12.5) years.

Most of the participating experts were involved with the surgery itself (67/71, 94%), decision making (eg, if patient is fit for surgery; 64/71, 90%), preoperative assessment (63/71, 89%), and cardiac intensive care unit (63/71, 89%). Some respondents also were involved with long-term follow-up of the patient (8/71, 11%) and in other ways (7/71, 10%), such as acute and chronic pain management and perioperative echocardiography.

#### Defining the Term “Postoperative Complication”

Comments were received from 50 experts on how they would define the term “Postoperative Complication” in cardiac surgery. The definitions emerging from Round 1 of the study were then used in the Round 2 questionnaire to reach consensus on a single definition.

All proposed definitions focused on different impacts of complications on the patient, institution, and surgery itself (eg, delayed recovery, impact on patient’s quality of life, and hospital length of stay). Hence, for simpler analysis, these statements were analyzed thematically [24] and categorized under themes based on the definitions that the experts offered. For example, the concept of “An unplanned adverse event occurring after cardiac surgery that may be caused or compounded by the surgical process” included statements such as “The event can be unplanned,” “The event must be harmful or unfavorable,” “The complication must be present following cardiac surgery, specifically,” and “The event must occur after surgery and is unlikely to occur if the patient did not have the surgery.” These common themes were then grouped and synthesized under common characteristics of the complications such as “unplanned,” “adverse event,” “cardiac surgery,” and “surgery.” All characteristics and themes can be found in Table 1.

**Table 1:** How experts voted for each characteristic that defines the term “complication after cardiac surgery” (N=38).

Theme	Complication characteristic	Results, n (%)
The event can have an impact on patient’s survival or quality of life and longevity.	Affects quality of life	35 (92)
The complication must be present following cardiac surgery, specifically.	Following cardiac surgery, specifically	33 (87)
The event must occur after surgery and is unlikely to occur if the patient did not have the surgery.	Due to surgical process	33 (87)
The event must be harmful or unfavorable.	Adverse event	28 (74)
The event can have an impact on hospital length of stay.	Delay in hospital discharge	28 (74)
Due to the event, the patient might have to stay in the hospital for longer and can adversely affect rapid recovery to good health.	Delay in recovery	28 (74)
The event can be expected but unplanned.	Unplanned	27 (71)
The event can be unexpected.	Unexpected	23 (61)

The responses for each definition were then mapped onto each characteristic to find what the experts deemed important to define what constitutes a postoperative complication following cardiac surgery, which could then be used for conducting Round 2 of the Delphi study.

#### Usefulness of Classifying Postoperative Complications

Responses to the question as to whether they thought it is useful to define and classify postoperative complications for cardiac surgery were provided by 51 experts. Of these 51 experts (Table 2), 23 (45%) thought it was “Extremely useful,” and 20 (39%) thought it is “Very useful.” Combining these percentages, based on the predetermined consensus level of 70%, it can be concluded that the experts have reached the consensus that it is very useful to classify postoperative complications for cardiac surgery, with a consensus level of 84%.

**Table 2:** Experts’ opinions on the usefulness of classifying postoperative complications following cardiac surgery (N=51).

Usefulness	Results, n (%)
Extremely useful	23 (45)
Very useful	20 (39)
Moderately useful	5 (10)
Slightly useful	2 (4)
Not at all useful	1 (2)

The experts provided various reasons why they thought it is useful to classify postoperative complications for cardiac surgery, which included improving audit and quality measurement, helping with planning and management, risk management and communications, and helping to improve research in the field. Some of the participants responded in the following ways when asked to explain why defining and classifying complications is useful:

Classification may help to understand causative factors and allocation of resources in prevention.

Expert R1.P56

This [classification of complications] could then be used to good effect in discussions with patients and families as they would gain consistent information from various members of the multi-disciplinary team.

Expert R1.P13

Categorising complications would be useful] to facilitate [...] research and to target therapies appropriately to prevent or decrease incidence.

Expert R1.P61

#### Categories of Postoperative Complications

Overall, 48 experts stated how many categories postoperative complications should have. Most of the respondents wanted 3 to 5 grades to categorize complications: Of the 48 respondents, 16 (33%) voted for 3 grades, 12 (25%) voted for 4 grades, and 14 (29%) voted for 5 grades. Some (26/48, 54%) also named the categories they offered, and it became clear that respondents offered the following variations as a common answer:

Mild/Moderate/ Severe

None/Mild/Moderate/Severe

Mild/Moderate/Severe/Death

None/Mild/Moderate/Severe/Death

All 26 experts who provided categories included mild/moderate/severe as the category combination. This means that consensus was reached that the categories for postoperative complications for cardiac surgery will be classified as “Mild,” “Moderate,” and “Severe.” Since many respondents offered “Death” as a separate class, the experts were asked to decide whether to add that to the categories in Round 2 of the study. Since no complication would be categorized as “None,” this was not added to the categories.

#### Defining the Categories of Postoperative Complications

Experts also provided possible definitions for each category that they proposed. To analyze the suggested definitions, the thematic analysis, explained in detail in the *Qualitative Analysis* section, focused on characteristics that each complication category could have. Like in the *Defining the Term “Postoperative Complication”* section, the characteristics provided by experts for each category of complications were collated so that similar characteristics were merged into one, and unique characteristics were left in their initial form [14]. The final list of characteristics proposed by the experts was as follows: effect on overall length of stay in hospital, effect on final outcome, length of the complication, clinical relevance, impact on the patient, occurrence of the complication, therapeutic intervention required, and impact on the institution

These factors were then related to a level of complication. For example, the question “What is the effect on overall length of stay in hospital?” was converted into “No consequential effect on overall length of stay” for the Mild level of complication, “Some effect on overall length of stay” for the Moderate level of complication, and “Extended length of stay” for the Severe level of complication. These statements were then used in Round 2 of the Delphi study so experts could vote on which characteristics were most important to define each complication category.

### Delphi Study Round 2

#### Development of the Questionnaire

The Round 2 survey (see Multimedia Appendix 2) of the Delphi study was sent to the same societies and contact list from the Scottish cardiac centers as described in the *Identification of Experts* section. To take part in Round 2, the experts were not required to have taken part in Round 1 of the study, as per the “all rounds” approach [15]. Just like in Round 1, the experts had to answer the filter question to make sure they were eligible to participate.

The aims of Round 2 of the study were to reach consensus regarding the following:

1. How do the experts define what constitutes a “postoperative complication following cardiac surgery” based on the responses from Round 1 of the study?
2. Should death be included in the categories of complications?
3. How do experts define each category of complications based on the characteristics collated from Round 1 of the study?

The choices for answers for the questions were collated based on the results of Round 1 of the study. Just like in Round 1, descriptive statistics were used to analyze the opinions of experts, using frequencies of responses for questions that were not open-ended. If the frequency of a response was 70% or higher, the experts were deemed to have reached consensus on this particular response.

Round 2 of the questionnaires were sent on June 2, 2020, and a reminder was sent on June 16, 2020. The survey was open for 4 weeks (closed on June 30, 2020).

Overall, 46 experts took part in the survey, and 37 of them finished the survey. As done in the previous round, we also included responses from participants who partially completed the survey in this round.

#### Experts’ Definition of What Constitutes “Postoperative Complications Following Cardiac Surgery”

Experts voted for each characteristic (see the *Defining the Term “Postoperative Complication”* section) to define what constitutes a complication after cardiac surgery. Consensus was reached that all characteristics (Table 1), apart from “Unexpected,” should be included in the final definition.

Combining these characteristics into a sentence resulted in the following definition:

A complication following cardiac surgery is an unplanned adverse event that occurs following cardiac surgery that can cause delay in recovery, cause delay in hospital discharge, and affect patient’s quality of life and is likely to happen due to the surgical process.

#### Including “Death” in the Classification of Postoperative Complications

Of 37 experts, 31 (84%) thought that “Death” should be included in the classification of postoperative complications. As a result, consensus was reached that the complications should be categorized in 4 levels: “Mild,” “Moderate,” “Severe,” and “Death.”

#### Defining the “Mild,” “Moderate,” and “Severe” Complication Categories

Based on the proposed characteristics that were collated from experts’ responses (described in the *Defining the Categories of Postoperative Complications* section), consensus was reached on definitions for “Mild” complications (Table 3). Hence, a complication following cardiac surgery is classified as “Mild” if the complication has the following characteristics: The complication has no consequential effect on the final patient outcome (28/37, 76%), and the complication has a minimal impact on the patient (27/37, 73%).

Similarly, as shown in Table 4, a complication following cardiac surgery is classified as “Severe” if the complication is potentially life-threatening (34/37, 92%), there is a consequential or long-standing impact on the patient (31/37, 84%), or a notable amount of intervention is required due to this complication (26/37, 70%).

The experts did not reach consensus on the definition for “Moderate” complications due to none of the characteristics receiving 70% or more of the votes (Table 5). However, one could argue that the definition of moderate is known, as it is neither mild nor severe. This is further discussed in the *Limitations* section.

**Table 3:** Characteristics of “Mild” complications (N=37).

Characteristic	Results, n (%)
Minimal impact on patient	28 (76)
No consequential effect on final outcome	27 (73)
No or only short-term clinical relevance	19 (51)
No or small amount of intervention required	19 (51)
No notable effect on overall length of stay	17 (46)
Mildly debilitating	7 (19)
Common	7 (19)
Minimal impact on institution	6 (16)
Lasting 1 week to 1 month	4 (11)

**Table 4:** The characteristics of “Severe” complications (N=37).

Characteristic	Results, n (%)
Potentially life-threatening	34 (92)
Consequential or long-standing impact on the patient	31 (84)
Notable amount of intervention required	26 (70)
Extended length of stay	25 (68)
With sustained relevance and life-limiting	25 (68)
Severely debilitating	21 (57)
Lasting 3 months to 1 year	7 (19)
Notable or long-standing impact on institution	5 (14)
Uncommon	2 (5)

**Table 5:** The characteristics of “Moderate” complications (N=37).

Characteristic	Results, n (%)
Some effect on overall length of stay	23 (62)
Acutely important but less clinical consequence long-term	22 (59)
Some intervention required	22 (59)
Some effect on final outcome	20 (54)
Moderately debilitating	19 (51)
Limited impact on patient	18 (49)
Lasting 1 month to 3 months	4 (11)
Less common	4 (11)
Limited impact on institution	4 (11)

Finally, to understand the experts’ understanding of which specific complications could fall into the established complication categories, experts were also asked to provide examples for each proposed complication level, examples of which were hemodynamic instability as a “Mild” complication, atrial fibrillation as a “Moderate” complication, and acute renal failure as a “Severe” complication. A list of examples of complications and how they were categorized by experts can be found in Multimedia Appendix 3. However, since there is currently no single nomenclature for surgical complications, unlike for clinical diagnosis (ie, the International Statistical Classification of Diseases-10), the classifications can vary, especially in the “Moderate” group. Hence, the list of complications and their categories presented in Multimedia Appendix 3 should be interpreted with caution.

## Discussion

### Principal Findings

We present the results of a pilot Delphi study that aimed to define and categorize complications following cardiac surgery. The study reached a consensus on the following: It is useful to define and categorize complications following cardiac surgery, how the complications following cardiac surgery are defined, and how the complications following cardiac surgery are classified.

The experts justified the usefulness of defining and categorizing surgical complications following cardiac surgery by stating it could help with audit and quality control, planning and management, risk management and communication, and research.

Consensus was reached on the characteristics of postoperative complications, and hence the following definition was formed:

*A complication following cardiac surgery is an unplanned adverse event that occurs following cardiac surgery that can cause delay in recovery, cause delay in hospital discharge, and affect patient’s quality of life and is likely to happen due to the surgical process.*

In the Clavien-Dindo classification system, complications were defined as “any deviation from the normal postoperative course,” and conditions that are inherent to the procedure and are expected were termed to be “sequelae” [26]. However, the definition from this Delphi study provides a more precise explanation of a complication. Also, as the Clavien-Dindo definition was created for general surgery, the definition presented in this study makes an important point that the Clavien-Dindo definition does not: A complication following cardiac surgery is an event that is unlikely to happen without surgery, specifically in our case, cardiac surgery. When it comes to the definition of “sequelae,” it can be argued that some adverse events following surgery can be expected, especially with existing and emerging preoperative prediction models. With improved data collection in electronic health records, more models predicting complications following surgery can be developed, meaning that many complications can be predicted and monitored on a real-time basis. Various studies have been published to predict fluid requirement [27], septic complications [28], hypotensive episodes [29], and clinical deterioration in general [30].

This study achieved consensus on how to categorize complications following cardiac surgery and how the categories are defined. It was agreed that the categories should be “Mild,” “Moderate,” “Severe,” and “Death.” According to the experts, a “Mild” complication is a complication that has no consequential effect on the final patient outcome and has minimal impact on the patient. The experts agreed that a “Severe” complication is a complication that is potentially life-threatening, requires a notable amount of intervention, and has a consequential or long-standing impact on the patient.

### Limitations

#### Study Sample

In Round 1 and Round 2 of the study, 51 and 37 experts completed the study, respectively. According to publications discussing the Delphi method, both rounds of the study had a sufficiently large sample size, as it does not depend on statistical power but rather on group dynamics for coming to consensus among experts. Hence, an expert panel usually consists of 10 to 30 experts [31]. Furthermore, since this was an e-Delphi study, it can be expected that the experts were not influenced by one another, as the respondents did not know what other respondents had said; therefore, the group dynamic came through each individual from analysis of experts’ responses.

As seen from the results of the study, most experts were cardiac anesthetists and intensivists; however, no cardiac surgeons took part in the study. Historically, the decision as to whether a patient will be operated upon is primarily made by the surgeon. Understanding surgeons’ views on defining and classifying complications in cardiac surgery would be useful. Hence, we have involved surgeons in an ongoing study regarding system requirements for a clinical decision support predicting complications. However, 90% of the participants in this study were involved with decision making, which is common with the creation of preassessment clinics, where decisions about patient care are made by multidisciplinary teams [32].

Although this is a pilot study that aimed to develop a classification system for complications in cardiac surgery, in future work, a more international panel of experts is needed to increase the impact of the classification system. Although experts within the European Association of Cardiothoracic Anaesthesiology and Intensive Care were invited, the majority of the professional societies were UK-based societies, which explains the lack of responses from international experts. Since the standards in cardiac surgery are common internationally [32], it is likely that results would be similar; however, the consensus would be more representative and more reliable to be put into practice. In addition, the societies were mostly related to cardiac anesthesia; only one (The Society for Cardiothoracic Surgery) was specific to cardiac surgeons. This explains why no cardiac surgeons took part in the study. However, it can be expected that, if surgeons took part in this study, the results would be similar due to growing interest in investigating postoperative outcomes other than mortality and an interest by both surgeons and anesthetists in improving patient outcomes beyond survival [33]. Hence, in our future study, cardiac centers will be contacted directly to allow for a more international panel, and more efforts will be directed toward recruiting more cardiac surgeons to participate.

#### Defining “Moderate” Complications

No consensus on the definition of “Moderate” complication was reached. Delphi studies do not always reach consensus on all aspects of the study [34]. Categorization decisions are often made based on the extreme categories rather than on the middle category [35]. This has been addressed with, for example, the American Society of Anesthesiologists (ASA) Classification [36], in which there is no “moderate” category. Historically, there have been concerns about the subjectivity of the ASA status [37], and the same problem can occur with our complication classification. To categorize complications appropriately, actions and consequences of each category need to be considered. With a “Mild” complication, some medicines might have to be administered, for example for urinary retention, but in general, no notable action that requires time and resources is needed. With a “Severe” complication, whether it is kidney failure or a stroke, dialysis or thrombectomy, respectively, might be needed. Both interventions are time-consuming and resource-intensive. When it comes to the moderate category, however, it is uncertain whether it is more on the “Mild” or “Severe” side. On one hand, it is generally unclear regarding what action needs to be taken; on the other hand, it provides the users with a spectrum of categories and therefore the possibility to offer more nuance to the problem. As shown by Mayhew et al [37], for the ASA physical status classification, providing example cases for each classification improved objectivity and reduced variability in classification. Hence, we also asked experts to provide examples for each category. However, further work is needed to provide examples; hence, it important to keep in mind that for personalized use, each complication, regardless of which category it falls into, needs an individual treatment approach, depending on the patient’s current state and medical history.

### Conclusion

Using the Delphi method, this pilot study shows cardiac anesthetists’ and cardiac intensivists’ requirements for a standardized definition and classification of postoperative complications in cardiac surgery. Standardization of complication identification, recording, and reporting in cardiac surgery could help the development of future quality benchmarks, clinical audits, care quality assessment, resource planning, risk management, performance comparisons or communication, and research.

## Appendix

Multimedia Appendix 1 Delphi study Round 1 questionnaire.

Multimedia Appendix 2 Delphi study Round 2 questionnaire.

Multimedia Appendix 3 Examples of postoperative complications and their proposed classifications as "Mild," "Moderate," or "Severe.".

## Abbreviations

ASA: American Society of Anesthesiologists

## Data Availability

The data sets generated or analyzed during this study are available from the corresponding author on reasonable request.
