# Supplementary material for: Definition and Classification of Postoperative Complications After Cardiac Surgery: Pilot Delphi Study
Source: JMIR Perioper Med. 2022 Oct 12;5(1):e39907. doi: 10.2196/39907 (PMC9607909; doi:10.2196/39907)
Supplement: Multimedia Appendix 1 [file periop_v5i1e39907_app1.docx]

### Multimedia Appendix 1. Delphi Study Round 1 Questionnaire

Demographics and Expertise

1. Are you in any way involved with cardiac surgery patients? (Can be preoperatively, intra-operatively, and/or postoperatively.)

- Yes
- No

1. What is your country of residence?
2. What is your speciality?

- Cardiac Anaesthetist
- Cardiac Surgeon
- Cardiac Critical Care
- Other (Please specify)

1. How long have you worked in this field (in years)?
2. What stages of cardiac surgery are you involved in?

- Pre-operative assessment
- Decision making (e.g., if patient is fit for surgery)
- The surgery itself
- ICU
- Long-term follow-up of the patient
- Other (Please explain)

Defining Postoperative Complications

1. How would you define the term “postoperative complication” in cardiac surgery? Please bring examples to explain.
2. How useful do you think it is to classify postoperative complications for cardiac surgery? Please explain your answer.

- Extremely useful
- Very useful
- Moderately useful
- Slightly useful
- Not at all useful

Classification of Postoperative Complications

1. In order to classify postoperative complication in cardiac surgery, how many grades should there be?
2. Based on the number of grades suggested, how would you define each of these grades?
3. Please provide an example of a complication for each of the suggested levels.
4. Is there anything else you would like to comment on the topic of postoperative complications?
