# Supplementary material for: Definition and Classification of Postoperative Complications After Cardiac Surgery: Pilot Delphi Study
Source: JMIR Perioper Med. 2022 Oct 12;5(1):e39907. doi: 10.2196/39907 (PMC9607909; doi:10.2196/39907)
Supplement: Multimedia Appendix 2 [file periop_v5i1e39907_app2.docx]

### Multimedia Appendix 2. Delphi Study Round 2 Questionnaire

1. Are you in any way involved with cardiac surgery patients? (Can be preoperatively, intra-operatively and/or postoperatively.)
2. From the selection below, please choose how postoperative complications after cardiac surgery should be defined. Multiple options are possible if combination of definitions is deemed important.

- An unplanned adverse event occurring after cardiac surgery that may be caused or compounded by the surgical process.
- An unplanned adverse event arising as a result of cardiac surgery, which was otherwise unlikely to have occurred in the same period.
- Any adverse event that impairs a patient’s physical, cognitive, psychological, or emotional function and quality of life.
- Any deviation from the ideal recovery pattern after cardiac surgery.
- Unexpected, or expected but unwanted, outcome of cardiac surgery which notably delays recovery from the procedure compared to the desired outcome or leads to the patient failing to derive the intended benefits of surgery.
- Any event resulting from surgery which lengthens the patient’s stay in hospital or reduces their quality of life beyond normal.
- Any unplanned clinical event that leads to a delay in hospital discharge or requires additional treatment or intervention to mitigate or reverse the event.
- Any deviation of any physiological system which adversely affects rapid recovery to good health.
- An event which may have an impact on patient’s survival or quality and longevity.

1. Based on your opinion, should death be included in the grading of postoperative complications? Please explain your answer.

- Yes
- No

1. For Mild Complication the definition should be (multiple options are possible):

- No notable effect on overall length of stay
- No notable effect on final outcome
- Lasting 1 week – 1 month
- No or only short-term clinical relevance
- Mildly debilitating
- Common
- No or small amount of intervention required
- Minimal impact on patient
- Minimal impact on institution

1. For Moderate Complication the definition should be (multiple options are possible):

- Some effect on overall length of stay
- Some effect on final outcome
- Lasting 1-3 months
- Acutely important, but less clinical consequence long term
- Moderately debilitating
- Less common
- Some intervention required
- Limited impact on patient
- Limited impact on institution

1. For Severe Complication the definition should be (multiple options are possible):

- Extended length of stay
- Potentially life-threatening
- Lasting 3 months – 1 year
- With sustained relevance and life-limiting
- Severely debilitating
- Uncommon
- Notable amount of intervention required
- Notable or long-standing impact on the patient
- Notable or long-standing impact on institution

1. If you have any comments regarding the topic of postoperative complications following cardiac surgery, please write them below.
