# Supplementary material for: Definition and Classification of Postoperative Complications After Cardiac Surgery: Pilot Delphi Study
Source: JMIR Perioper Med. 2022 Oct 12;5(1):e39907. doi: 10.2196/39907 (PMC9607909; doi:10.2196/39907)
Supplement: Multimedia Appendix 3 [file periop_v5i1e39907_app3.docx]

### Multimedia Appendix 3. List of Complications and Their Categories

| Complication | Category |
| --- | --- |
| Cardioverted | Mild |
| Defibrillated | Mild |
| Haemodynamic instability | Mild |
| Low Systemic Vascular Resistance State | Mild |
| Pyrexia of Unknown Origin | Mild |
| Sternal Resuturing | Mild |
| Urinary Retention | Mild |
| Acute Cholecystitis | Moderate |
| Atrial Fibrillation | Moderate |
| Bleeding Peptic Ulceration | Moderate |
| Delayed Sternal Closure | Moderate |
| Fasciotomy | Moderate |
| Femoral Artery Embolectomy | Moderate |
| Femoral Wound Infection | Moderate |
| Haemothorax Requiring Drain | Moderate |
| Heel Pressure Sore | Moderate |
| Ischaemic Limb | Moderate |
| Leg wound dehiscence | Moderate |
| Leg Wound Infection | Moderate |
| Leg Wound Leak | Moderate |
| Low Cardiac Output | Moderate |
| Minitracheostomy | Moderate |
| Nasogastric Feeding | Moderate |
| Pacing Dependence Delaying Discharge | Moderate |
| Paravalve Leak | Moderate |
| Perioperative Myocardial Infarction | Moderate |
| Permanent Pacemaker | Moderate |
| Postoperative Elevated Creatinine | Moderate |
| Prolonged ileus | Moderate |
| Psychosis Requiring Treatment | Moderate |
| Pulmonary Infection Requiring Antibiotics | Moderate |
| Return to the theatre: re-operation for bleeding or tamponade | Moderate |
| Return to Theatre: Re-operation for graft problems | Moderate |
| Return to theatre: re-operation for other cardiac problems | Moderate |
| Return to theatre: re-operation for valvular problems | Moderate |
| Return to theatre: Sternum Resuturing (sterile) | Moderate |
| Reventilated | Moderate |
| Sacral Pressure Sore | Moderate |
| Secondary Haemorrhage | Moderate |
| Sternal Dehiscence | Moderate |
| Sternal Wound Leak | Moderate |
| Superficial Wound Infection | Moderate |
| Thigh Wound Infection | Moderate |
| Thigh Wound Leak | Moderate |
| Total Parenteral Nutrition | Moderate |
| Unstable Angina | Moderate |
| Urinary Tract Infection | Moderate |
| Ventricular Fibrillation/Tachycardia | Moderate |
| Acute Renal Failure | Severe |
| Adult Respiratory Distress Syndrome | Severe |
| Amputation | Severe |
| Cardiac Arrest | Severe |
| Deep Sternal Wound Infection | Severe |
| Hepatic Failure | Severe |
| Left Ventricular Wall Dissection | Severe |
| Mesenteric Infarction | Severe |
| Multiorgan Failure | Severe |
| Open Tracheostomy | Severe |
| Paraparesis | Severe |
| Percutaneous Tracheostomy | Severe |
| Permanent Stroke | Severe |
| Reopening Requiring Cardiopulmonary Bypass | Severe |
| Required Laparotomy | Severe |
| Septicaemia | Severe |
| Severe Heart Failure | Severe |
| Severe Pulmonary Oedema | Severe |
| Transient Stroke | Severe |
